# The role of non-coding RNAs in extracellular vesicles in breast cancer and their diagnostic implications

**DOI:** 10.1038/s41388-023-02827-y

**Published:** 2023-09-05

**Authors:** Mark Samuels, William Jones, Benjamin Towler, Charlotte Turner, Stephen Robinson, Georgios Giamas

**Affiliations:** Department of Biochemistry and Biomedicine, School of Life Sciences, University of Sussex, JMS Building, Falmer, Brighton, BN1 9QG UK

**Keywords:** Breast cancer, Cancer microenvironment

## Abstract

Breast Cancer (BC) is the most common form of cancer worldwide, responsible for 25% of cancers in women. Whilst treatment is effective and often curative in early BC, metastatic disease is incurable, highlighting the need for early detection. Currently, early detection relies on invasive procedures, however recent studies have shown extracellular vesicles (EVs) obtained from liquid biopsies may have clinical utility. EVs transport diverse bioactive cargos throughout the body, play major roles in intercellular communication and, importantly, mirror their cell of origin. In cancer cells, EVs alter the behaviour of the tumour microenvironment (TME), forming a bridge of communication between cancerous and non-cancerous cells to alter all aspects of cancer progression, including the formation of a pre-metastatic niche. Through gene regulatory frameworks, non-coding RNAs (ncRNAs) modulate vital molecular and cellular processes and can act as both tumour suppressors and oncogenic drivers in various cancer types. EVs transport and protect ncRNAs, facilitating their use clinically as liquid biopsies for early BC detection. This review summarises current research surrounding ncRNAs and EVs within BC, focusing on their roles in cancer progression through bi-directional communication with the microenvironment and their diagnostic implications.

**Figure:**
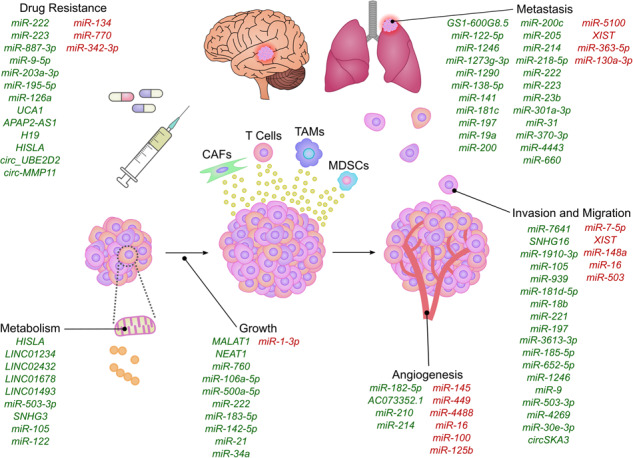
The role of EV ncRNAs in breast cancer. A representation of the different EV ncRNAs involved in tumourigenic processes in breast cancer. Pro-tumourigenic ncRNAs displayed in green and ncRNAs which inhibit oncogenic processes are shown in red.

The role of EV ncRNAs in breast cancer. A representation of the different EV ncRNAs involved in tumourigenic processes in breast cancer. Pro-tumourigenic ncRNAs displayed in green and ncRNAs which inhibit oncogenic processes are shown in red.

## Introduction

Accounting for 11.7% of cancer diagnoses and 6.9% of cancer deaths, breast cancer (BC) is the most common cancer worldwide, with 1 in 8 women developing BC in their lifetime [1]. Differences in molecular features and expression identifies 4 subtypes of BC: luminal A, luminal B, human epidermal growth factor receptor 2 (HER2)-positive, and triple-negative (TNBC) [2]. Defining BC subtype allows tailoring of treatment to the patient, increasing the chances of tumour eradication, and preventing recurrence or therapy resistance. Unfortunately, metastatic BC remains incurable, and treatment aims to relieve symptoms and prolong survival [3]. Many high-income countries have established screening programmes involving mammograms, ultrasounds, and biopsies to identify early BC, increasing the chances of successful treatment [4]. Unfortunately, these tests can be time consuming, unpleasant, and invasive, discouraging individuals from undergoing screening. It is therefore vital to develop new methods that are less invasive, fast, and accurate to improve the early diagnosis of BC.

## Extracellular vesicles

Extracellular vesicles (EVs) are small, non-replicative, lipid bilayer-delimited particles, released by nearly all cell types in every organism [5]. EVs contain bioactive cargo, including lipids, metabolites, nucleic acids, and proteins, and can be categorised based on their biogenesis. Exosomes (typically 30–150 nm) are produced through the endosomal pathway during maturation of early endosomes to late endosomes/multivesicular bodies (MVBs) where the inward budding of the MVB membrane produces intraluminal vesicles (ILVs). ILVs are then released as exosomes when MVBs fuse with the plasma membrane [6]. Microvesicles (~50–1000 nm) are produced when the plasma membrane undergoes outwards budding. Other EV subtypes include apoptotic bodies, an important player in apoptosis [7], and oncosomes which are secreted by cancer cells to aid in tumour growth and the development of the tumour microenvironment (TME) [5].

EVs can alter the behaviour of surrounding cells, which is particularly important in cancer as cancer cells use EVs to change the phenotype of surrounding cells in the TME, promoting growth, metastasis, and therapy resistance. EVs have been proposed as a diagnostic tool as they can shield their cargo from nucleases and proteases in biological fluids and can reflect the phenotype of their cell of origin. Importantly, EVs are found in almost all biological fluids, allowing for easy and non-invasive collection [5].

## Non-coding RNAs

Traditionally, RNAs were thought primarily to enable transfer of instructions from DNA to ribosomes to produce proteins. However, recently, many new types of RNA have been discovered and categorised into different classes depending on size and function. MicroRNAs (miRNAs) are approximately 22 nucleotides (nt) in length and are loaded into the RNA-induced silencing complex (RISC) to induce translational repression and/or degradation of target RNAs containing complementary sequences. PIWI-interacting RNAs (piRNAs) are 24–31 nt long and bind to the PIWI family of proteins, allowing epigenetic regulation of chromatin through transposon silencing [8]. Long non-coding RNAs (lncRNAs) are over 200 nt and regulate transcription, nuclear organisation, proteins, and can act as miRNA decoys to regulate gene expression, whereas circular RNAs (circRNAs) are closed loops of RNA with roles in gene expression regulation [9, 10]. Each of these non-coding RNA (ncRNA) classes can work as oncogenes or tumour suppressors, contributing to regulation of cancers, including BC [11].

### Regulation of ncRNA sorting into EVs

**Fig. 1:**
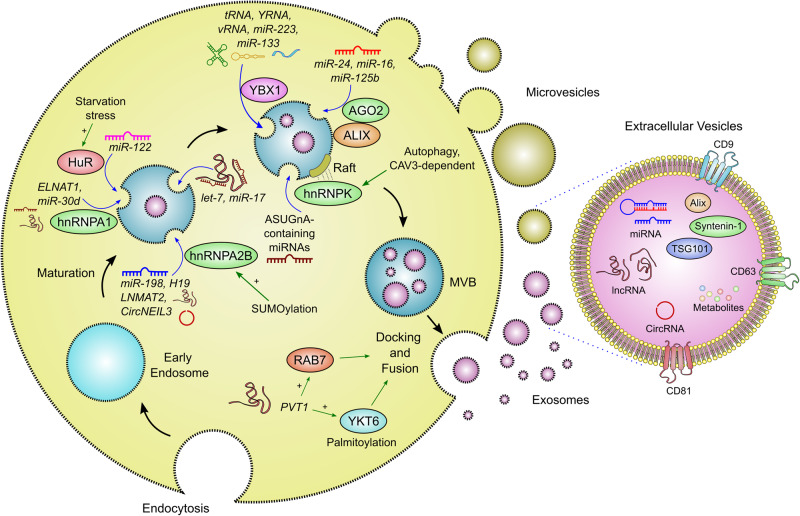
Mechanisms governing the loading of ncRNAs into extracellular vesicles. An array of molecular mechanisms are implicated in the regulation of loading a diverse repertoire of ncRNAs, including lncRNAs, miRNAs, circRNAs and tRNA into extracellular vesicles. These appear to involve crucial roles for RNA binding proteins (RBPs) such as hnRNP family members, HuR and YBX1 recognising specific motifs in target RNAs. Loading of ncRNAs into these structures is a tightly regulated process with the RBPs involved controlled by specific cellular conditions, including cellular stress, providing a dynamic system to regulate cell-cell communication under different environmental conditions. ncRNAs themselves have also been suggested to directly influence extracellular vesicle formation, loading of miRNAs containing complementary seed sequences, and docking and therefore appear to lie central to extracellular vesicle biology.

Concise regulation of the ncRNA composition of EVs is critical to maintain homoeostasis, drive angiogenesis, and aid in the response to external stimuli including cellular stress. The ncRNA composition of EVs is not simply a reflection of cellular composition and therefore, there is active regulatory processing governing the loading of ncRNAs into EVs (Fig. 1).

Perhaps the most well-studied within this context is the heterogeneous nuclear ribonucleoprotein (hnRNP) family. For instance, hnRNPA2B1 directly interacts with GAGG motifs, termed EXOmotifs [12], within target RNAs including the miRNA *miR-198* [12] and the lncRNAs *H19* [13] and *LNMAT2* [14], whilst it negatively regulates *miR-503* sorting into endothelial cell (EC) EVs [15]. CircRNAs, including *circNEIL3*, are also loaded into EVs by hnRNPA2B1 [16]. Interestingly, the role of hnRNPA2B1 in EV loading is sensitive to post-translational regulation such as SUMOylation [12] together with O-GlcNAcylation in response to oxidative stress [17]. SUMOylation of hnRNPA1 is important for the regulation of the lncRNA *small nucleolar RNA host gene 16 (SNHG16)* (otherwise known as *ELNAT1*) and its packing into EVs [18]. These mechanisms present a beautifully dynamic system for controlling ncRNA secretion through EVs (Fig. 1).

Other members of the hnRNP family have also been implicated in controlling ncRNA sorting into EVs. For example, hnRNPC1 is important for *miR-30d* loading into EVs [19] whilst Santangelo et al. identified a GGCU motif in miRNAs such as *miR-3470a* and *miR-194-2-3p* which was important for hnRNPQ-mediated loading [20, 21]. Additionally, Robinson et al. demonstrated that hnRNPK localises to MVBs in a membrane-raft-dependent mechanism where it recruits specific miRNAs carrying an AsUGnA motif in prostate cancer cells [22]. Interestingly, hnRNPK interacts with the autophagy machinery, in a mechanism defined as LC3-Dependent EV Loading and Secretion (LDELS) [23]. Deficiencies in the LC3-conjugating machinery change the EV ncRNA landscape in a caveolin 1-dependent manner [24]. This mechanism is largely independent of the ESCRT machinery and may reveal a stress-sensitive mechanism for regulating EV content. Therefore, despite their predominantly nuclear localisation, there is overwhelming evidence for nuclear-independent roles for these family members and future work is crucial to understand the molecular mechanisms that govern their shuttling between cellular compartments.

Alongside hnRNP family members, several other RNA binding proteins (RBPs) regulate ncRNA loading into EVs. For example, Lupas La has been demonstrated to drive specific loading of *miR-122* into CD63-enriched vesicular high density (vHD) bodies in MDA-MB-231 BC cells [25]. HuR, a potent post-transcriptional regulator, also orchestrates EV-mediated miRNA export, including *miR-122*, during starvation stress in hepatic cells with this activity sensitive to HuR ubiquitination [26]. HuR has also been implicated in regulating the packaging of *miR-1246* into EVs through an interaction with an AUUUU motif [27]. Furthermore, RBP YBX1 has been implicated in the sorting of ncRNAs, including transfer RNAs (tRNAs), YRNAs, viral RNAs (vRNAs) and *miR-223* [28, 29], and in regulating *miR-133* loading into EVs to promote fibroblast angiogenesis and mesenchymal-endothelial transition [30]. Fragile X messenger ribonucleoprotein 1 (FMR1), another RBP, was also demonstrated to chaperone miRNAs containing an AAUGC motif for internalisation in a process involving the hepatocyte growth factor regulated tyrosine kinase substrate [31]. In addition to those discussed here numerous other RBPs including insulin like growth factor 2 mRNA binding protein 1 (IGF2BP1) [32], major vault protein (MVP) [33], MEX3C1 [34] and annexin A2 (ANXA2) [15, 35] have been implicated in regulating the sorting of ncRNAs into EVs.

The role of protein argonaute-2 (Ago2), a crucial component of the RISC, has also been explored. Whilst there is debate over the presence of Ago2 in EVs which may be driven by technical factors during EV isolation, KRAS mutational status or culture conditions (reviewed in [36]), phosphorylation of Ago2 on Ser 387 has been implicated in the loading of specific miRNAs into EVs in a KRAS and mitogen-activated protein kinase kinase (MEK) activity dependent mechanism [37]. Evidence towards Ago2-miRNA complex loading into EVs was also provided by Lavello et al. where a direct interaction between the adaptor protein Alix, itself involved in EV biogenesis, and Ago2 was presented [38]. They demonstrated that Alix depletion in human liver stem-like cells resulted in a decrease in several miRNAs, including *miR-24*, *miR-16* and *miR-125b* and inferred that the Alix-Ago2 interaction was important for EV loading [38]. Moreover, an Ago2 knockout mouse model, demonstrated that those miRNAs that are most sensitive to Ago2 depletion are amongst the most highly exported miRNAs [39]. These data provide a foundation for the further work that are required to ascertain the direct role of Ago2-mediated miRNA loading into EVs.

RNA-RNA interaction also influences the RNA content of EVs. For example, Ahadi et al. observed lncRNAs in EVs from prostate cancer cells were enriched in miRNA seed sequences that were similarly enriched within EVs, including members of the *let-7* and *miR-17* families [40], suggesting these interactions may drive EV presence. The sequence specificity of RNA for localisation to the lipid rafts was also explored using RNA aptamers and identified four motifs that were enriched in both raft-localised aptamers and in pro-tumoral EV-enriched miRNAs, which strikingly included the EXOmotif CCCU previously identified by Villarroya-Belri et al. [12, 41]. In addition to templated RNA motifs, RNA modifications may also participate in ncRNA loading into EVs with untemplated terminal nucleotide additions differentiating between EV enriched miRNAs (3’ uridylated) vs cell enriched (3’ adenylated) miRNAs from B-cells [42]. Interestingly, alongside being actively loaded into EVs, lncRNAs may also regulate the formation of EVs [43, 44]. For instance, *PVT1* was shown to promote the docking of MVBs by influencing RAB7 expression and localisation together with promoting palmitoylation of YKT6 and its co-localisation with vesicle-associated membrane protein 3 (VAMP3) [43]. It is conceivable that by influencing the formation of EVs, ncRNAs themselves could control EV content through additional mechanisms other than direct RNA-RNA interactions.

Once the EVs reach the recipient cell membrane they can be internalised either by endocytosis, receptor-ligand interactions, or direct fusion with the cell membrane. The proportion of released ncRNA that is functional is unclear, however, it is likely that the interactions with the RBPs which may drive their initial inclusion into EVs are crucial for their subsequent function within the recipient cell.

## The role of ncRNAs in breast cancer

Across the following sections, the roles of specific EV-carried ncRNA’s in different oncogenic processes will be comprehensively discussed. The ncRNA’s discussed are all summarised in Table 1.

**Table 1:** The roles of ncRNAs in EVs in BC.

Process	ncRNA	Target	Function	Reference
Invasion and metastasis	*miR-200*	*–*	EMT suppression promoting lung metastasis	[90]
	*miR-181d-5p*	*CDX5*	CAF EVs downregulate CDX5 and HOXA5, enhancing EMT	[47]
	*miR-7-5p*	*RYK*	Increased EMT transcription factor expression	[51]
	*miR-18b*	*TCEAL7*	Induction of EMT through SNAIL activation	[48]
	*miR-1910-3p*	*MTMR3*	Inhibition of apoptosis and autophagy activating NF-κB and WNT signalling, promoting migration	[49]
	*miR-221*	*PTEN*	Enhances EMT and metastasis	[54]
	*miR-7641*	*–*	Promotes invasiveness and metastasis	[50]
	*miR-105*	*ZO-1*	Destruction of tight junctions in ECs, increasing vascular permeability	[56]
	*miR-939*	*VE-cadherin*	Increases endothelial permeability, facilitating trans-endothelial migration of BC	[57]
	*SNHG16*	*PPAPDC1A*	Sponging of *miR-892b* to regulate PPAPDC1A, promoting EMT, migration and invasion	[55]
	*miR-214*	*TFAP2C*	Stromal EVs containing *miR-214* promote metastasis following IL-6/STAT3 signalling activation	[60]
	*GS1-600G8.5*	*–*	Increased BBB permeability due to downregulation of tight junction proteins	[61]
	*miR-181c*	*PDPK1*	BBB breakdown through changes in actin dynamics	[62]
	*miR-363-5p*	*PDGFB*	Inhibits migration and lymph node metastasis	[63]
	*miR-222*	*PDLIM2*	Activation of NF-κB signalling and promotion of migration	[64]
	*miR-130a-3p*	*RAB5B*	Inhibition of invasion and lymph node metastasis	[65]
	*miR-370-3p*	*FBLN5*	Activation of NF-κB signalling and promotion of migration and stemness	[69]
	*miR-193b*	*RAB22A*	Promotion of growth and metastasis	[66]
	*miR-19a*	*PTEN*	Astrocyte EVs downregulate PTEN in BC cells promoting outgrowth of brain metastatic cells	[71]
	*miR-1290/miR-1246*	*FOXA2*	Activation of astrocytes and *miR-1290* promotes CNTF signalling, activating progression of brain metastases	[74]
	*miR-301a-3p*	*TIMP-2*	Astrocyte interactions supporting the formation of a tumour-supportive environment in the brain	[76]
	*miR-503*	*–*	*XIST* downregulation in BC promotes *miR-503* upregulation in EVs which promote M1-M2 conversion in microglia	[77]
	*miR-19a*	*PTEN*	Activation of AKT signalling and promotion of bone metastasis in ER + BC	[80]
	*miR-218*	*YY1, INHBB*	Regulation of collagen deposition in osteoblasts, promoting osteolysis and facilitating bone metastasis	[81]
	*miR-1273g-3p*	*BMP3*	*SNHG3* regulates Bone marrow MSC differentiation in bone metastasis through *miR-127*3g*-3p* and BMP3 regulation	[82]
	*miR-4443*	*TIMP2*	Upregulation of MMP-2 in the liver and primary tumour, facilitating liver metastasis	[83]
	*miR-122-5p*	*Syndecan-1*	Increased cell mobility and metastatic potential	[85]
	*miR-200c, miR-141*	*–*	FOXP3 induces *miR-200c* and *miR-141* upregulation of release from BC cells, promoting distal metastasis	[87]
	*miR-5100*	*CXCL12*	PGRN knockout TAMs inhibit migration and EMT through *miR-5100* release and inhibition of CXCL12 signalling	[88]
	*miR-3613-3p*	*SOCS2*	CAF-derived EVs promote BC invasion and metastasis through *miR-3613-3p*	[93]
	*miR-370-3p*	*CYLD*	Fibroblast activation through BC EVs containing *miR-370-3p* promotes lung metastasis	[89]
	*miR-185-5p, miR-652-5p, miR-1246*	*–*	BC cells promote CAF transition through miRNAs. The CAFs then promote migration of BC cells	[94]
	*miR-9*	*E-cadherin*	BC cells release EVs containing *miR-9* to promote CAF formation, CAFs secrete *miR-9* promoting BC metastasis	[95]
	*miR-146a*	*TXNIP*	*miR-146a* from BC cells promotes CAF transition through TXNIP regulation as well as EMT and growth in BC	[96]
	*miR-16, miR-148a*	*–*	Abrogation of FAK signalling in CAFs increases *miR-16* and *miR-148a* in EVs, reducing BC metastasis	[97]
	*miR-503-3p, miR-4269, miR-30e-3p*	*–*	ELK3 expression in lymphatic ECs promotes miRNA packaging in EVs to promote metastasis in BC	[98]
	*miR-503*	*CCND2, CCND3*	EVs from vascular ECs inhibit tumour growth and invasiveness in response to chemotherapy	[99]
	*miR-205, miR-31*	*UBE2N/Ubc13*	MSC-derived EVs suppress migration through UBE2N/Ubc13 regulation in non-committed BC cells but promote primary tumour progression	[100]
	*miR-23b*	*MARCKS*	BM-MSCs promote dormancy through regulation of MARCKS in BC cells	[101]
	*miR-660*	*KLHL21*	TAM-derived EVs promote metastasis in BC through *miR-660*-mediated activation of NF-κB signalling	[68]
	*miR-138-5p*	*KDM6B*	EVs from BC promote M2 polarisation in macrophages through KDM6B downregulation, promoting lung metastasis	[92]
	*circSKA3*	*–*	Promotes invasiveness through EV transfer from invasive BC cells to less invasive BC cells	[102]
	*RN7SL1*	*–*	RN7SL1 EVs activate inflammatory responses in immune cells in response to NOTCH-MYC signalling	[103]
Growth	*MALAT1*	*–*	BC EVs containing MALAT1 induce cell proliferation	[105]
	*miR-500a-5p*	*USP28*	CAF-derived *miR-500a-5p* in EVs promotes increased proliferation and metastasis	[109]
	*miR-760*	*ARF6*	M2-derived CCL18 upregulates *miR-760* in BC cells which is released in EVs to promote proliferation in BC cells	[107]
	*miR-197*	*PPARG*	BC stem cell EVs containing *miR-197* promotes proliferation and EMT in other BC cells	[86]
	*NEAT1*	*miR-141-3p*	*NEAT1* in EVs from BC sponges *miR-141-3p*, regulating KLF12 expression, promoting growth and metastasis	[106]
	*miR-106a-5p*	*–*	*miR-106a*-5p from MSCs accelerates cancer progression. *HAND2-AS1* inhibits *miR-106a*-*5p*	[108]
	*miR-1-3p*	*GLIS1*	CAF-derived *miR-1-3p* targets GLIS1, promoting tumour spheroid formation	[110]
	*miR-21, miR-34a*	*–*	MSC-derived EVs promote BC growth	[114]
	*miR-222*	*PTEN*	BC EVs containing *miR-222* cause PTEN downregulation in macrophages, promoting M2 polarisation and facilitating tumour growth in vivo	[111]
	*miR-183-5p*	*PPP2CA*	BC EVs containing *miR-183-5p* suppress PPP2CA expression in macrophages, increasing growth and metastasis	[112]
	*miR-142-5p, miR-183-5p, miR-222-3p*	*PTEN*	EVs from ECs promote M2 polarisation in macrophages, promoting tumour growth	[113]
Angiogenesis	*miR-182-5p*	*CMTM7*	EVs from BC containing *miR-182-5p* promoted angiogenic behaviour in HUVEC cells though EGFR/AKT signalling	[115]
	*AC073352.1*	*YBX1*	EVs from BC containing *AC073352.1* increased angiogenic behaviour in HUVECs	[116]
	*miR-210*	*Ephrin A3 and PTP1B*	Desferrioxamine-mediated induction of hypoxia in BC promotes *miR-210* transfer to ECs inducing angiogenesis	[117]
	*miR-214*	*ATM*	Endothelial cell-derived EVs promote endothelial cell migration through suppression of cell cycle arrest	[118]
	*miR-145*	*IRS1*	EVs from BC with increased Ca^2+^ levels induce angiogenesis though PI3K/Akt signalling	[119]
	*miR-4488*	*CX3CL1*	Mitochondrial calcium uniporter (MCU) enhances angiogenesis through EV *miR-448*8 downregulation	[120]
	*miR-16*	*VEGF*	MSC-derived EVs inhibit HUVEC tube formation and migration through VEGF regulation	[121]
	*miR-100*	*mTOR*	MSC-derived EVs inhibit HUVEC proliferation and migration through VEGF and HIF1α regulation	[122]
	*Pro-angiogenic miRNAs*	*–*	DHA suppresses angiogenesis via increasing anti-angiogenic EVs from BC cells and decreasing pro-angiogenic miRNAs in EVs from BC cells	[123]
	*miR-125b*	*–*	Wharton’s Jelly MSC EVs upregulate *miR-125b* in BC cells inhibits HIF1 activation, reducing angiogenesis	[124]
Drug resistance	*miR-222*	*ERα, PTEN*	Resistant BC cells and stromal cells transfer *miR-222* to BC cells inducing resistance to tamoxifen, docetaxel and adriamycin	[128–130]
	*miR-222, miR-223*	*–*	MSCs release EVs to promote quiescence and carboplatin resistance in BC	[131]
	*miR-887-3p*	*BTBD7*	EVs from BC could be uptaken by other BC cells producing resistance to doxorubicin, cisplatin and fulvestrant	[132]
	*miR-9-5p, miR-203a-3p, miR-195-5p*	*ONECUT2*	Chemotherapeutics cause EV release in BC cells, promoting stemness, NOTCH1, SOX9 and NANOG expression	[133]
	*miR-126a*	*–*	MDSC-derived EVs induce angiogenesis and Th2 cell responses in response to doxorubicin	[134]
	*UCA1*	*–*	Induction of tamoxifen resistance in tamoxifen sensitive BC cells	[135]
	*AGAP2-AS1*	*–*	Induction of resistance to Trastuzumab in BC	[136]
	*H19*	*–*	Doxorubicin resistance through transfer of EVs to sensitive BC cells	[137]
	*NEAT1*	*miR-141-3p*	*NEAT1* transfer to BC cells promotes resistance to cisplatin, paclitaxel and 5-FU through KLF1 regulation	[106]
	*Circ_UBE2D2*	*miR-200a-3p*	Resistance to tamoxifen in BC cells by sponging *miR-200a*-3p	[139]
	*Circ-MMP11*	*miR-153-3p*	Sponging *miR-153-3p* regulates Anilin, promoting Lapatinib resistance in BC cells	[140]
	*miR-134*	*–*	Regulation of Bcl-2 increasing sensitivity to cisplatin	[141]
	*miR-770*	*STMN1*	Overexpression increases Doxorubicin sensitivity. *miR-770* packaged into EVs and transported to TAMs, promoting M1 polarisation	[142]
	*miR-342-3p*	*ID4*	MSC-derived EVs transfer *miR-342-3p* to BC tissues, suppressing migration and increasing sensitivity to doxorubicin, fluorouracil and cisplatin	[143]
Metabolism	*circCARM1*	*miR-1252-5p*	BC stem cell-derived EVs cause increased expression of glycolytic enzymes in tumours	[144]
	*HISLA*	*PHD2*	*HISLA* from TAMs stabilizes HIF1α by binding to PHD2, preventing degradation, driving metabolic reprogramming	[138]
	*miR-503-3p*	*DACT2*	Macrophage EVs regulate DACT2, upregulating WNT signalling to promote glycolysis and reduce oxidative phosphorylation	[145]
	*SNHG3*	*miR-330-5p*	CAF-derived SNHG3 suppresses *miR-330-5p* in BC cells, upregulating pyruvate kinase and glycolytic activity	[146]
	*miR-105*	*MXI1*	BC EVs downregulate the MYC antagonist MXI1 in CAFs, leading to metabolic reprogramming	[147]
	*miR-122*	*PKM2*	EVs from BC with *miR-122* suppress glucose metabolism in primary tumours and brain and lung tissues	[148]
	*miR-122*	*PKM2*	EVs from BC cells alter insulin signalling in pancreatic islet β cells, increasing systemic glucose levels	[149]

This table displays the different ncRNAs identified as having a role in BC progression through EV-mediated communication, the targets of the ncRNAs and the effect on cells.

### Invasion and metastasis

**Fig. 2:**
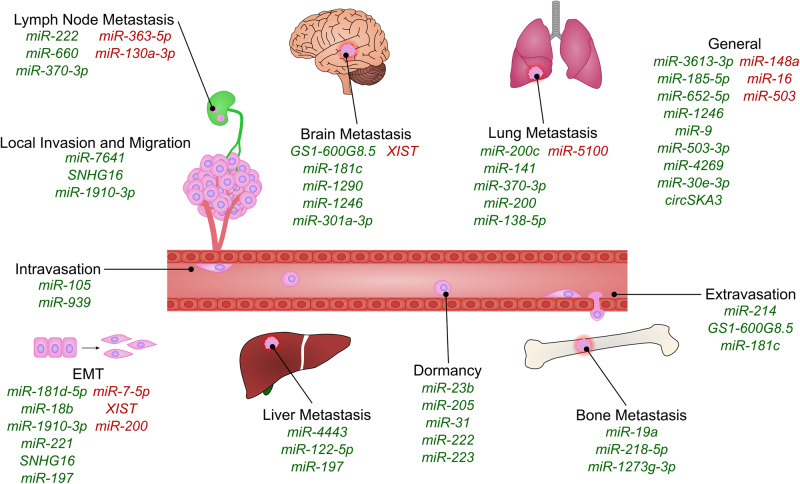
The role of ncRNAs in invasion and metastasis. A figure showing the different EV ncRNAs involved in the different processes of invasion and metastasis. ncRNAs promoting processes are displayed in green and ncRNAs which inhibit processes are shown in red.

Cancers cause morbidity and mortality through the invasion of local structures and the secondary spread to distant organs (metastasis). Metastasis is a multi-stage process and the leading cause of treatment failure and mortality in cancer. Bi-directional communication between BC and the TME is crucial for both processes. Additionally, the formation of a metastatic niche in distal organs creates a supportive microenvironment for secondary tumours to form. Extensive research has been performed into the role of EV ncRNAs in this process and this section will describe how EV-contained ncRNAs are involved in each step (Fig. 2).

### Local invasion

During local invasion, BC cells break though the basement membrane into the surrounding tissue and extracellular matrix. Epithelial-Mesenchymal transition (EMT) is an important part of this process where epithelial cancer cells utilise a complex developmental program to turn off epithelial genes and upregulate EMT transcription factors including those of the SNAIL, TWIST and ZEB families to promote a mesenchymal phenotype [45]. The in vivo relevance of this process is heavily debated due to tissue-specific roles of the different EMT transcription factors and research showing their role in the aggressiveness of non-epithelial tumours [46]. EMT genes may therefore exert effects through altering cell plasticity and de-differentiation in BC rather instead of simply through classical EMT [45]. Therefore, research into EMT in BC is diverse, suggesting many different roles of EMT-related ncRNAs.

Cancer-associated fibroblast (CAF)-derived EVs containing *miR-181d-5p* enhance BC aggressiveness through targeting CDX2 and downregulating HOXA5 [47]. This process enhances EMT through the regulation of N-cadherin, SLUG, SNAIL1, TWIST1, ZEB1 and ZEB2. A *miR-181d-5p* inhibitor reversed the effect of CAF EVs on EMT in BC cells and restored HOXA5 expression. *miR-18b* is upregulated in EVs derived from CAFs compared to normal fibroblasts (NFs) and promotes EMT through transcription elongation factor A like 7 (TCEAL7) inhibition, activating SNAIL through nuclear factor-kappa B (NF-κB) [48]. The authors showed that this pathway also promoted metastasis in a xenograft mouse model, suggesting that CAFs may be a useful target in BC treatment.

Another group identified *miR-1910-3p* as an important regulator of metastasis and autophagy by targeting myotubularin related protein 3 (MTMR3), activating NF-κB signalling. Overexpression of *miR-1910-3p* causes upregulation of SLUG and TWIST and a reduction in E-cadherin. Inhibition of *miR-1910-3p* reduces N-cadherin, Vimentin, SLUG, TWIST, B-cell lymphoma 2 (Bcl-2) and Proliferating cell nuclear antigen (PCNA) expression. Functionally, overexpression of *miR-1910-3p* promoted proliferation and migration in vivo and EVs from *miR-1910-3p* overexpressing cells increased invasiveness when taken up by recipient cells. Mechanistically, this was due to inhibition of apoptosis and induction of autophagy, which they suggested was through inhibition of MTMR3 expression and activation of NF-κB and Wnt signalling [49].

Shen et al. identified *miR-7641* as a promoter of BC metastasis by using EVs from metastatic MDA-MB-231 or HCC-1937 cells, or non-metastatic MCF-7 cells to treat MCF-7 and primary BC cells in transwell invasion assays and wound-healing assays. EVs from metastatic cell lines promoted invasion and migration in non-metastatic cell lines, with *miR-7641* upregulated in the cells and EVs of invasive cells. Additionally, *miR-7641* overexpression increased the migration and invasion of both MCF-7 and MDA-MB-231 cells, whereas inhibitors had the opposite effect. This effect was also confirmed in vivo [50]. Separately, in a bioinformatic screen, Liang et al. found *miR-7-5p* is upregulated in EVs from less invasive BC cells [51]. Comparison of MDA-MB-231 and MCF-7 cell lines showed that EVs from highly invasive BC are more likely to induce migration than EVs from less invasive BC lines, with *miR-7-5p* mimics promoting E-cadherin expression and inhibiting N-cadherin expression. Overall, they showed that *miR-7-5p* targets receptor like tyrosine kinase (RYK), reducing c-Jun N-terminal kinase (JNK) phosphorylation and inhibiting EMT [51].

Protease-activated receptor 2 (PAR2) is overexpressed in TNBC [52] and contributes to migration through matrix metalloproteinase (MMP)-2 induction [53]. Upon PAR2 activation, TNBC cells package *miR-221* into EVs, promoting EMT through Phosphatase and tensin homologue (PTEN) targeting, causing AKT/NF-κB activation [54]. lncRNAs have also been shown to play a role in EMT. Xia et al. demonstrated that EVs carrying *SNHG16* significantly enhance migration, invasion and EMT in BC cells [55], through the regulation of PPAPDC1A by *SNHG16*, resulting in *miR-892b* sponging.

### Intravasation

The entry of BC cells into the bloodstream or lymphatic system is crucial for dissemination to distant organs. Communication between BC and ECs is critical to enable angiogenesis and intravasation. The cells must then survive in the circulation before they undergo extravasation and form secondary tumours.

Transfer of *miR-105* from BC cells to ECs occurs in an EV-mediated fashion [56]. *miR-105* is upregulated in metastatic BC cell lines compared to primary breast cell lines and even more so in EVs, indicating selective packaging of *miR-105* into EVs. *miR-105* targets Zonula occludens-1 (ZO-1), reducing the barrier function of the ECs, increasing vascular permeability. Additionally, overexpression of *miR-105* in non-metastatic BC cells is sufficient to induce metastasis in xenograft mouse models. Another group found *miR-939* to have an important role in the downregulation of VE-cadherin, destroying the barrier function of ECs, showing upregulation of *miR-939* in TNBC and a correlation with poor prognosis and lymph node metastasis [57].

### Extravasation

During extravasation, BC cells rely on adherence to the walls of the vasculature, mediated by changes in cell-cell adhesion proteins and bidirectional communication with ECs to exit the lumen of blood vessels and colonise new tissue. *miR-214* is a pro-metastatic miRNA in TNBC [58] with well-described roles in increasing cell motility, promoting extravasation and increasing survival to anoikis [59]. Orso et al. demonstrated that BC cells induce the expression of *miR-214* in CAFs through interleukin-6 (IL-6)/Signal transducer and activator of transcription 3 (STAT3) signalling. Subsequently *miR-214* is packaged into EVs and taken up by BC cells, activating a pro-metastatic program [60].

Another study established a highly metastatic BC cell line through in vivo selection [61]. EVs from these cells were internalised by brain microvascular ECs, reducing trans-endothelial electrical resistance, and increasing blood-brain barrier (BBB) permeability. The lncRNA *GS1-600G8.5* was upregulated in the highly metastatic cell line compared to parental cells and *GS1-600G8.5* silencing abrogated the BBB permeability phenotype. *miR-181c* also increases BBB permeability, enabling cancer cell migration through the BBB [62]. Phosphoinositide-dependent protein kinase-1 (PDPK1) downregulation by *miR-181c* promotes alterations to actin dynamics and localisation due to a reduction in phosphorylated cofilin, enabling trans-BBB migration.

### Formation of metastases

#### Formation of lymph node metastases

Axillary lymph node metastasis is a critical step in the progression of BC and an important prognostic marker in early BC. Due to the modest false negative rate of sentinel lymph node biopsy, a study examined the expression profiles of circulating EVs for biomarkers of lymph node metastasis. The authors showed *miR-363-5p* was significantly downregulated in EVs from patients with lymph node metastasis where its expression levels correlated with improved survival [63]. Functionally, they determined that *miR-363-5p* may have a tumour suppressor role, inhibiting colony formation, migration and invasion. Platelet derived growth factor subunit B (PDGFB) was identified as a target of *miR-363-5p*, suggesting a potential mechanism for the tumour suppressive effect.

*miR-222* is an oncogenic miRNA that is highly expressed in BC with lymph node metastasis. Ding et al. found that tumour cell *miR-222* overexpression led to increased EV *miR-222* that could be transferred to other cells. *miR-222* targets the tumour suppressor gene PDLIM2, promoting activation of NF-κB signalling, whilst *miR-222* inhibition decreased MDA-MB-231 invasiveness [64]. *miR-130a-3p* has been shown to inhibit migration and invasion through RAB5B regulation, and is downregulated in circulating EVs in BC patients, whilst overexpression in BC stem cells (BCSCs) inhibits migration and proliferation through G0/G1 arrest. Additionally, low levels of *miR-130a-3p* correlated with lymph node metastasis, suggesting it may be a useful indicator of lymph node metastases and a potential therapeutic target [65]. RAB22A is an important regulator of intracellular trafficking, and upregulation is associated with lymph node metastasis in BC. RAB22A is a target of *miR-193b* and RAB22A knockdown or *miR-193b* overexpression decrease EV release and reduce the ability of EVs to promote proliferation [66].

Tumour-associated macrophages (TAMs) are key regulators of angiogenesis, metastasis and immunosuppression in BC, particularly in the preparation of the pre-metastatic niche and participation in pro-tumourigenic signalling pathways [67]. TAM-derived EVs can also promote metastasis, with one group finding that *miR-660* in TAM derived-EVs promotes lymph node metastasis in BC through targeting Kelch like family member 21 (KLHL21) and activating the NF-κB p65 signalling pathway [68]. Another study explored the role of *miR-370-3p* in BC and found it is highly expressed in EVs, with the level of expression positively correlating with lymph node metastasis. Overexpression of *miR-370-3p* in BC cells promotes mobility and proliferation whereas knockdown has the opposite effect [69].

#### Formation of brain metastases

Cancer cells must adapt in order to survive at distal sites, through transcriptome changes and crosstalk with the TME [70]. In a landmark study, Zhang et al. [71] showed that PTEN was downregulated in brain metastases by miRNAs in astrocyte derived-EVs and rescued by the depletion of PTEN-targeting miRNAs in astrocytes, reducing brain metastasis in vivo. They identified increased C-C motif chemokine 2 (CCL2) secretion, recruiting Iba1+ myeloid cells, further promoting proliferation of brain metastatic tumour cells. This study highlights the importance of communication between metastatic tumour cells and the new microenvironment, lending evidence to the seed and soil hypothesis [72], and provides new therapeutic avenues to explore in the inhibition of BC metastasis. Additionally, tGLI1, a transcription factor known to promote brain metastases [73], has been shown to activate astrocytes by EV-mediated transfer of *miR-1290* and *miR-1246*, inhibiting FOXA2 and promoting Ciliary neurotrophic factor (CNTF) cytokine secretion, priming the brain metastatic niche [74].

Morad et al. explored the role of EVs in brain metastasis in TNBC using a brain-seeking variant of MDA-MB-231 cells, generated through sequential passaging in nude mice [75]. Interestingly, they found non-canonical Cdc42-dependent clathrin-independent carrier/GPI-AP-enriched compartments (CLIC/GEEC) endocytosis to be important in astrocyte uptake of BC EVs. Proteomics showed upregulation of surface markers known to be cargo of the CLIC/GEEC endocytic pathway in brain-seeking EVs. The EVs reduced expression of Tissue inhibitor of metalloproteinases 2 (TIMP2) in astrocytes, increasing their migration through *miR-301a-3p*. Analysis of clinical data also showed that *miR-301a-3p* levels correlate with decreased survival [76].

In another study, profiling lncRNAs from brain metastatic breast tumours revealed downregulation of *X* *inactive specific transcript* (*XIST*), whilst in xenografts *XIST* expression inversely correlated with brain metastasis [77]. Mechanistically, *XIST* downregulation promotes EMT and activates c-Met, promoting stemness. Additionally, EVs from *XIST* downregulated cells promoted M1-to-M2 conversion in microglia through *miR-503* regulation. Finally, the authors showed that fludarbine treatment of *XIST* low BC cells effectively inhibited brain metastasis in mouse models, demonstrating an interesting new synthetic lethality therapeutic approach.

#### Formation of bone metastases

Bone metastases occur in most metastatic BC patients [78], leading to complications including bone fracture, severe pain and bone marrow infiltration [79]. To investigate why oestrogen receptor positive (ER+) BC has a preference for bone metastasis, Wu et al. characterised the transcriptomes of bone-tropic and non-bone-tropic BC cells, identifying *miR-19a* and integrin binding sialoprotein (IBSP) upregulation in bone-tropic ER+ BC cell EVs. These EVs induced osteoclastogenesis in osteoclasts, creating a favourable environment in the bone. The authors also identified *miR-19a* and IBSP overexpression induced bone metastasis in an ectopic MCF-7 mouse model, whereas neither could promote it alone [80], further demonstrating the importance of ncRNAs in EVs in priming new metastatic sites in BC.

*miR-218-5p* is significantly upregulated in bone metastatic BC, but not brain metastatic BC [81]. A study showed EVs from *miR-218* overexpressing MDA-MB-231 cells significantly downregulated type I collagen expression and deposition by osteoblasts compared to control EVs when injected into mice, contributing to the adaptation of the bone metastatic niche by promoting osteolysis to facilitate bone metastasis [81]. The lncRNA *SNHG3* is a key regulator of bone marrow mesenchymal stem cell (MSC) osteogenesis in BC bone metastasis. *SNHG3* regulates the *miR-1273g-3p*/bone morphogenetic protein 3 (BMP3) axis to promote osteogenesis, and *SNHG3* levels correlate with increased bone metastasis. BMP3 expression positively correlates with *SNHG3* and is regulated by EV-contained *miR-1273g-3p* [82].

#### Formation of liver metastases

Liver metastasis is another common occurrence in advanced BC. One group found that EV-contained *miR-444*3 promotes BC metastasis through TIMP2 downregulation and the upregulation of MMPs, whilst overexpression of *miR-444*3 in non-invasive BC cells led to liver metastasis [83]. Syndecan-1, which also associates with BC metastasis [84], is suppressed by *miR-122-5p*. *miR-122-5p* is enriched in liver cell-derived EVs and increases with liver injury. These EVs increase BC cell motility through Syndecan-1 suppression [85].

Another study found that BCSC-derived EVs increased the proliferation of MDA-MB-231 and SUM149PT cells in vitro and in vivo where they also promote liver metastasis. These EVs were shown to deliver *miR-197*, targeting *PPARG* mRNA and promoting EMT and proliferation in the cells [86].

#### Formation of lung metastases

*miR-200c* and *miR-141* are associated with lung metastasis in BC [87]. In Foxp3 heterozygous Scurfy mutant mice, breast tumours form spontaneously and metastasise to the lung. *miR-200c* and *miR-141* levels in plasma increase throughout tumour progression and this pattern is consistent with human samples. Zhang et al. suggest that EV-contained *miR-200c* and *miR-141* are regulated by the FOXP3-KAT2B axis and may be useful biomarkers for BC metastasis [87].

TAMs also play a role in promoting metastasis. Progranulin knockout in mice was shown to reduce lung metastasis with Progranulin positive BC cells [88]. *miR-5100* was upregulated in Progranulin knockout TAMs and the group suggested that, through *miR-5100*-mediated inhibition of the CXCL12/CXCR4 axis, this reduced the invasiveness and metastatic potential of BC cells due to the pivotal role of CXCL12/CXCR4 axis in cancer cell migration, proliferation and gene regulation. Overall, the authors suggest Progranulin downregulation in TAMs promotes upregulation of *miR-5100* in EVs, reducing lung metastasis in BC.

CAF induction by BC cells can occur through several different signalling pathways. Ren et al. described how *miR-370-3p* from BC cell EVs induces fibroblast activation through CYLD regulation [89]. They found EVs from multiple BC cell lines induced activation of NFs which were then able to enhance migration, invasion and EMT of BC cells. *miR-370-3p* was responsible for the activation of fibroblasts through downregulation of CYLD, altering NF-κB signalling, ultimately promoting lung metastasis in an in vivo model.

*miR-200* is found in EVs derived from metastatic 4T1 cells but not the poorly metastatic 4TO7 cell line [90] and *miR-200* family miRNAs suppress EMT through ZEB1 and ZEB2 regulation [91]. A study showed that EVs from MCF10CA1a cells could promote lung colonisation of MDA-MB-231 cells in immunocompromised mice via *miR-200*. MCF10CA1a cells readily form secondary tumours in nude mice, however MDA-MB-231 cells were reported to be less able to form lung metastases. This study showed the ability of metastatic cells to induce a metastatic phenotype in less metastatic BC cells via EVs, leading to local invasion and the colonisation of the lung [90]. Another group showed that BC-derived EVs contain *miR-138-5p* which is taken up by macrophages and promotes M2 polarisation through lysine demthylase 6B (KDM6B) downregulation, leading to promotion of lung metastasis [92].

#### Metastasis promoting roles of TME Cells

In addition to organ specific effects, CAFs have also been shown to generally promote metastasis. *miR-3613-3p* from CAF EVs promotes metastasis through regulating suppressor of cytokine signalling 2 (SOCS2) expression [93]. *miR-3613-3p* is upregulated in CAF EVs following education from BT474 and MCF-7 BC cells. The authors proposed that regulation of SOCS2 was essential to this mechanism and clinical data negatively correlated SOCS2 and *miR-3613-3p* expression in BC samples.

Another group found that *miR-185-5p*, *miR-652-5p*, and *miR-124*6 promoted CAF specialisation [94]. EVs from MDA-MB-231 cells promoted CAF transition, increasing invasiveness in breast epithelial cells. *miR-9* also plays a role in CAFs as BC EVs containing *miR-9* were shown to promote CAF formation, which was abrogated upon *miR-9* inhibition. Interestingly, *miR-9* from CAFs could promote invasiveness in BC cells through downregulating E-cadherin [95]. Yang et al. also showed that *miR-146a* was important in CAF activation. EVs from BC cells containing *miR-146a* promoted CAF transition as well as BC growth and EMT in nude mice. Thioredoxin interacting protein (TXNIP) was found to be a target of *miR-146a*, causing WNT activation in fibroblasts [96].

Focal adhesion kinase (FAK) signalling in CAFs may be involved in BC migration and metastasis. Ablation of FAK increases *miR-16* and *miR-148a* in EVs from fibroblasts which are then less able to promote metastasis than wild-type CAFs [97]. In addition to fibroblasts, lymphatic vessel endothelial cells (LECs) have been proposed as contributors to metastasis. ELK3 in LECs is proposed to be essential for the metastasis-promoting properties of LEC-derived EVs, with *miR-503-3p*, *miR-4269* and *miR-30e-3p* identified as key mediators [98]. Another study found that *miR-503* impairs tumour growth and invasiveness and was abundant in EVs released from vascular ECs. They found *CCND2* and *CCND3* were targets of *miR-503* and interestingly, chemotherapy increases release of *miR-503* in plasma [99].

MSC-derived EVs also play a role in BC where they induce dormancy and suppress metastasis through *miR-205* and *miR-31* [100]. Notably, this effect was only seen in parental MDA-MB-231 cells and not organotropic metastatic MDA-MB-231 sublines. In primary tumours, the EVs promoted growth in both parental and organ-specific metastatic lines, whereas they suppress metastasis through promoting dormancy in the parental cell lines. The authors suggested that UBE2N/Ubc13 regulation was involved in the process as it is a target of the miRNAs and silencing of UBE2N/Ubc13 also suppresses migration, invasion, and proliferation of BC cells. Overall, the authors showed that MSCs play a role in promoting dormancy in non-committed metastatic BC cells but do not reduce metastasis in committed BC cells.

Another study also found that bone marrow MSCs play a role in promoting dormancy in metastatic BC cells through suppression of proliferation, protecting cancer cells from chemotherapies [101]. They found that bone metastatic BC cells upregulated *miR-23b* and downregulated myristoylated alanine rich protein kinase C substrate (MARCKS), reducing cell cycle progression. The authors suggested that transfer of *miR-23b* from bone marrow MSCs to BC cells was responsible for the promotion of dormancy in the BC cells.

CircRNAs are also implicated in tumour growth. *circSKA3* from EVs promotes cell growth and invasiveness, and higher levels of *circSKA3* correlate with increased potential to form large colonies [102]. *circSKA3* transfer occurs between different BC cells, enabling regulation of less invasive BC cells by more invasive BC cells. An interesting study found that stromal NOTCH-MYC signalling promoted the generation of unshielded *RN7SL1*-containing EVs. *RN7SL1* is normally shielded by the RBP SRP9/14, however when unshielded, it is transferred to immune cells, generating an inflammatory response, acting as a damage-associated molecular pattern (DAMP), activating retinoic acid-inducible gene I (RIG-I), increasing proliferation, metastasis and therapy resistance in BC [103]. These EVs increase myeloid/dendritic cell populations expressing maturation and activation markers in the spleen, however due to the complexity of the immune microenvironment, the mechanism was not elucidated.

### Cell growth

Uncontrolled cell growth is a fundamental hallmark of cancers, and is achieved through the activation of proliferative signalling and evasion of growth suppression signals [104]. A growing body of research demonstrates that EV cargoes derived from TME cells and BC themselves can promote proliferation in BC cells, with key studies linking specific ncRNA cargo with proliferation in BC.

*Metastasis associated lung adenocarcinoma transcript 1 (MALAT1)* is upregulated in BC and BC-derived EVs and associated with progression, where high levels correlate with shorter survival, whilst siRNA against *MALAT1* reduces cell proliferation [105]. The lncRNA *nuclear paraspeckle assembly transcript 1* (*NEAT1)* associates with lymph node metastasis and Ki-67 in BC and is overexpressed in serum EVs in BC patients. BC patient EVs promoted proliferation in MCF-7 and MDA-MB-231 cells, whereas healthy volunteer EVs did not, and this was reversed by *NEAT1* inhibition. Mechanistically, *NEAT1* is a sponge for *miR-141* which is frequently downregulated in BC, increasing tumourigenicity and contributes to metastasis and chemoresistance. By sponging *miR-141-3p*, *NEAT1* regulates Krüppel-like factor 12 (KLF12), promoting growth, chemoresistance and metastasis [106].

ADP-ribosylation factor 6 (ARF6) plays a role in CCL18 signalling in BC metastasis. A study found that CCL18 treatment increased ARF6 and p-AMAP1 expression, activating PI3K/Akt signalling. Interestingly, *miR-760*, which targets ARF6, was found to be highly expressed in EVs secreted from BC cells stimulated by CCL18. MCF-7 cells take up these EVs and become more proliferative and invasive, surprisingly, through *miR-760*-mediated upregulation of ARF6 and subsequent activation of PI3K/Akt signalling. Overall, this study showed that the M2-derived cytokine, CCL18 promotes upregulation of *miR-760* in EVs, resulting in proliferation, chemoresistance and metastasis [107].

*miR-106a-5p* is upregulated in TNBC compared to healthy tissue, where it associates with poorer prognosis [108]. MSCs release EVs containing *miR-106a-5p* which are taken up by TNBC cells. *HAND2-AS1*, an antisense RNA which inhibits *miR-106a-5p* expression and secretion from MSCs, is negatively correlated with tumour grade and downregulated in TNBC cells. In vivo, *HAND2-AS1* injection inhibited tumour growth in nude mice.

One study isolated CAFs and NFs from BCs and adjacent tissue and screened EVs from these cells for miRNAs. They found *miR-500a-5p* was highly expressed in CAFs and their EVs and upregulated in recipient BC cells after treatment with the EVs. The authors suggested that these EVs promote proliferation and metastasis through the downregulation of ubiquitin-specific peptidase 28 (USP28). In an in vivo model, CAFs overexpressing *miR-500a-5p* promoted increased tumour size in MDA-MB-231 xenografts [109]. Another study found that *miR-1-3p* was downregulated in BC tissue and that CAFs from surrounding tissue had reduced *miR-1-3p* in their EVs compared to NFs. CAF EVs were able to deliver *miR-1-3p* to BC cells and *miR-1-3p* overexpression in CAFs promoted suppression of tumour formation and metastasis in BC cells in a coculture system. Krüppel-like zinc-finger protein Gli-similar 1 (GLIS1) was suggested as the target of *miR-1-3p* and this was confirmed by a dual-luciferase reporter assay and overexpression of GLIS1 abrogated the effects of *miR-1-3p* on BC development [110].

BC cells also communicate with TAMs to promote growth. *miR-222* from adriamycin-resistant BC cells induces M2 polarisation in macrophages. A study showed this led to an increase in proliferation in vivo in *miR-222* overexpressing BC cells through targeting of PTEN in macrophages, which, in turn, activated Akt signalling, facilitating M2 polarisation and pro-tumour signalling [111]. Guo et al. showed that BC-derived EVs transferred *miR-183-5p* to macrophages, downregulating PPP2CA and increasing NF-κB signalling, leading to IL-1β, IL-6, and tumour necrosis factor alpha (TNF-α) expression in macrophages and a pro-inflammatory phenotype [112]. Interestingly, *miR-183-5p* knockdown in BC suppressed tumour growth and metastasis in a mouse model.

Another study found that endothelial-derived EVs promote tumour growth through induction of an M2-like phenotype in macrophages [113]. *miR-142-5p*, *miR-183-5p* and *miR-222-3p* are released in EVs from ECs which increase M2 signature gene expression. The authors suggest that targeting of PTEN by the miRNAs was responsible for the increase in the M2 markers, arginase-1 (ARG1) and transforming growth factor beta (TGF-β), promoting tumour growth. A comprehensive study of MSC-derived EVs used RNA sequencing, proteomics and lipidomics to analyse MSC EV cargo and found *miR-21* and *miR-34a* to have an important tumour supportive role by promoting proliferation in BC cells [114].

### Angiogenesis

Angiogenesis allows tumours to acquire sufficient oxygen and nutrients through the formation of neovasculature whilst facilitating metastases by promoting intravasation. The process is regulated through the balance of pro- and anti-angiogenic factors and the triggering of an “angiogenic switch” depending on the relative abundance of these factors. Tumour cells, particularly in hypoxic conditions, secrete significant quantities of pro-angiogenic factors, which act on ECs to induce angiogenic signalling in the existing vasculature, triggering angiogenic sprouting and new vessel formation. Transfer of biomolecules through EVs plays a role in angiogenic signalling, with EV-associated ncRNAs having both pro- and anti-angiogenic effects.

Overexpression of *miR-182-5p* in BC tissues correlates with poor patient prognosis, and transfection of *miR-182-5p* mimic into human umbilical vein endothelial cells (HUVECs) enhanced proliferation, migration and angiogenesis [115]. In this study, BC EVs delivered *miR-182-5p* to HUVECs, inducing the same angiogenic phenotype. Mechanistically, *miR-182-5p* reduces expression of CKLF like MARVEL transmembrane domain containing 7 (CMTM7) tumour suppressor, leading to activation of EGFR/AKT signalling and subsequent angiogenic signalling, which was confirmed in vivo [115].

Kong et al. performed a microarray analysis of BC tissues and analysis of The Cancer Genome Atlas (TCGA) data, identifying differentially expressed lncRNA’s in BC. Novel lncRNA *AC073352.1* is upregulated in tumour tissues and correlates with poor prognosis. Mechanistically, *AC073352.1* binds to YBX1 transcriptional activator to stabilise it and promote metastasis. Notably, YBX1 contributed to the packaging of *AC073352.1* into MDA-MB-231 EVs and the uptake of *AC073352.1*-carrying BC-derived EVs increased angiogenic activity in HUVECs [116].

Hypoxic conditions in a murine BC model increased EV secretion from 4T1 cells, with increased cellular and EV levels of *miR-210* reported after desferrioxamine-mediated induction of hypoxia inducible factor 1 alpha (HIF1α) signalling [117]. ECs treated with hypoxic 4T1 cell-derived EVs exhibited increased migration, capillary-like structure formation and proliferation compared cells treated with control EVs. Vascular remodelling proteins and *miR-210* targets, ephrin-A3 and PTP1B, were decreased within the TME of tumours treated with hypoxic EVs whereas vascular endothelial growth factor (VEGF) and Ki-67 levels were increased, demonstrating increased angiogenesis [117].

One study found endothelial cell-derived EVs promoted endothelial cell migration in a scratch wound assay, and significantly increased tubule length and sprouting in a Matrigel tubule formation assay. *miR-214* was upregulated 3-fold in EVs relative to cells, and EV-mediated induction of endothelial cell migration and tubule formation was dependent on the expression of *miR-214*, which suppresses cell cycle arrest through ATM downregulation [118].

There are also examples of anti-angiogenic ncRNAs in EVs. Increased calcium levels in MDA-MB-231 cells led to increased EV secretion, and EVs released from BC cells with A23187-treatment elevated intracellular Ca^2+^ levels significantly increased angiogenic activity in recipient HUVECs [119]. Concurrently, EVs from MDA-MB-231 cells treated with SKF96365 Ca^2+^ influx inhibitor had anti-angiogenic effects on recipient HUVECs, due to *miR-145* and *miR-449* upregulation. *miR-145* suppresses insulin receptor substrate 1 (IRS1), inhibiting pro-angiogenic PI3K/Akt and MAPK signalling, and IRS1 was downregulated in HUVECs following treatment with anti-angiogenic EVs. STIM1, an essential regulator of Ca^2+^ signalling, was downregulated in response to calcium depletion, leading to *miR-145* upregulation in BC cells, BC-derived EVs and the recipient HUVECs, ultimately decreasing angiogenesis [119].

Similarly, the mitochondrial calcium uniporter (MCU), a key Ca^2+^ channel implicated in the progression of multiple cancer types, reportedly enhances angiogenesis in the metastatic niche of BC through the downregulation of *miR-4488* in BC-derived EVs. MCU suppression in MDA-MB-231 cells led to the secretion of EVs that reduced liver metastasis and angiogenesis in vivo, and modulation of MCU expression in vitro altered the abundance of many miRNAs in EV cargo. A notable negative correlation between cellular MCU expression and EV *miR-4488* levels was reported, attributable to MCU-mediated negative selective sorting of miRNA to EV cargo. *miR-4488* suppresses angiogenesis by targeting CX3CL1 mRNA, and levels of *miR-4488* in serum EVs of TNBC patients were shown to be lower than in non-TNBC patients, highlighting the suppression of *miR-4488* sorting into EVs as a mechanism by which MCU expression might promote angiogenesis in the metastatic niche and contributes to a more aggressive disease phenotype [120].

MSCs reportedly have conflicting pro- and anti-tumourigenic roles. Multiple studies have identified that miRNAs carried by MSC EVs have anti-angiogenic effects. One such example identified downregulation of VEGF in 4T1 murine BC cells through the transfer of *miR-16* from MSC EVs. Accordingly, EVs from MSCs suppressed angiogenesis in vitro and in vivo [121]. Additionally, Pakravan et al. showed that EV-mediated transfer of *miR-100* from MSCs to MDA-MB-231 BC cells significantly reduces VEGF expression in recipient BC cells via *miR-100*-mediated mTOR downregulation and HIF1α suppression [122].

Omega-3 fatty acid docosahexaenoic acid (DHA), which has anti-cancer efficacy through the suppression of angiogenic factors, significantly decreases the levels of pro-angiogenic miRNAs and increases anti-angiogenic miRNAs in both MDA-MB-231 BC cells and EVs [123]. Additionally, Chang et al. explored the potential of using EVs from Wharton’s Jelly MSCs (WJ-MSCs) therapeutically, showing that WJ-MSC EVs reduce in vitro proliferation, sphere formation, migration and EMT in MDA-MB-231 cells and in vivo metastasis in a murine model. WJ-MSC-derived EVs significantly altered BC cell miRNA expression profiles, upregulating miRNAs associated with inhibited tumour development. The authors highlight *miR-125b* as significantly elevated in WJ-MSC EV cargo and upregulated in recipient BC cells and show that *miR-125b* directly regulates HIF1α, hypothesising that this inhibits HIF1 activation and subsequent downstream gene expression changes that drive proliferation, EMT and angiogenesis [124].

### Drug resistance

One primary challenge for successful treatment of most cancers remains the development of drug resistance. Many factors contribute to treatment resistance, including upregulation of efflux transporters, mutations, and physical inaccessibility of drugs to poorly vascularised tumour regions. EV-mediated transfer of ncRNAs from resistant cancer cells to sensitive cells is an important mechanism for propagation of tumour drug resistance. This topic has been reviewed deeply, in a 2021 systematic review exploring the role of EV-carried miRNAs in the induction of chemoresistance in multiple cancer types [125], and in recently published BC focused reviews by Weon Yi [126] and Rezaee et al. [127].

The transfer of many different ncRNAs from resistant tumour cells or stromal cells to sensitive BC cells induces resistance to BC chemotherapeutics. *miR-222* is transferred in EVs between drug-resistant and sensitive tumour cells, conferring resistance to common BC drugs including tamoxifen, docetaxel and adriamycin by downregulating ERα and PTEN in recipient cells [128–130], and EV-mediated transfer of *miR-222* and *miR-223* from MSCs to BC cells in the bone marrow induces BC cell dormancy, promoting quiescence and resistance to carboplatin in metastatic cells prior to recurrence [131].

One study examined EVs derived from MDA-MB-231 BC cells, finding that MCF-7, BT474 and HCC1937 BC cell survival under doxorubicin, cisplatin and fulvestrant treatment respectively is significantly increased following EV-mediated transfer of *miR-887-3p*, BTB domain containing 7 (BTBD7) suppression and Notch signalling activation in recipient cells. Notably, inhibition of *miR-887-3p* in MDA-MB-231 cells abrogated the EV-driven induction of drug resistance in recipient BC cells [132].

Treatment of naïve MDA-MB-231 BC cells with EVs from docetaxel- or doxorubicin-treated MDA-MB-231 cells induces stemness-associated genes like NOTCH1, SOX9 and NANOG. Several miRNAs were shown to be upregulated in “chemo-EVs”, including *miR-9-5p*, *miR-203a-3p* and *miR-195-5p*, which induce this stemness phenotype through inhibition of ONECUT2, a master regulator of cell fate. Importantly, in a xenograft mouse model, the induction of BC stemness by EV-carried miRNAs reduced tumour docetaxel sensitivity [133].

Treatment of 4T1 BC-bearing mice with doxorubicin led to induction of myeloid derived suppressor cells (MDSCs), reportedly promoting tumour growth, metastasis, angiogenesis and anti-inflammatory Th2 responses via EV-carried *miR-126a*. Inhibition of *miR-126a* increased the efficacy of chemotherapy against lung metastasis in 4T1 tumour-bearing mice through local angiogenic suppression. This exemplifies how BC tumours adapt to doxorubicin treatment, promoting lung metastasis through induction of angiogenesis and Th2 cell responses in the metastatic niche to facilitate BC cell survival [134].

LncRNAs such as *UCA1*, *APAP2-AS1* and *H19* have been separately shown to promote resistance to tamoxifen, trastuzumab and doxorubicin respectively [135–137]. Furthermore, the transfer of HIF1A stabilizing long noncoding RNA (*HISLA*) from TAMs reportedly increases BC resistance to chemotherapeutics through HIF-1α stabilisation and subsequent inhibition of apoptosis [138]. *Circ_UBE2D2* and *circ-MMP11* also promote tamoxifen and lapatinib resistance through *miR-200a-3p* and *miR-153-3p* sponging respectively in BC cells through EV-mediated lncRNA transfer from resistant to sensitive cells [139, 140].

ncRNAs can also enhance treatment sensitivity. One study showed downregulation of *miR-134* in EVs from an aggressive clonal variant (Hs578Ts(i)_8_) of Hs578T BC cells and significantly lower expression in patient breast tumour tissue compared to normal controls. Interestingly, overexpression of *miR-134* mimic in Hs578Ts(i)_8_ reduced expression of anti-apoptotic protein Bcl-2 and increased cisplatin sensitivity. The authors demonstrated that EVs from Hs578Ts(i)_8_ cells transfected with *miR-134* mimic promoted reduced aggressiveness and increased sensitivity to anti-heat shock protein 90 (HSP90) compounds through downregulation of signal transducer and activator of transcription 5B (STAT5B) and HSP90 in recipient TNBC cells [141].

Ectopic overexpression of *miR-770* in MDA-MB-468 and MDA-MB-231 cells significantly increased doxorubicin sensitivity in another study. EV packaging of *miR-770* and transfer to TAMs, wherein pro-inflammatory M1 polarisation is promoted, suppressed macrophage driven chemo-resistance in TNBC cells. Overexpression of *miR-770* significantly decreased stathmin 1 (STMN1) expression, increasing doxorubicin sensitivity and metastasis in both BC lines and, in a xenograft mouse model, *miR-770* promoted TNBC treatment sensitivity through the EV-mediated transfer of *miR-770* to TAMs, suggesting that TNBC loss of *miR-770* represents a key mechanism of chemo-resistance acquisition [142].

Finally, *miR-342-3p* is enriched MSC-derived EVs, which suppressed invasion and increased doxorubicin, fluorouracil and cisplatin sensitivity in MCF-7 cells in a study from Yu et al. Conversely, inhibition of *miR-342-3p* in SKBR-3 cells significantly increased invasion and migration and chemotherapeutic resistance. ID4 is an *miR-342-3p* target, and inhibitor Of DNA binding 4 (ID4) inhibition in BC cells increased MCF-7 chemo-sensitivity and suppressed tumour growth and EMT in vivo [143].

### Metabolism

Cellular metabolism in most healthy cells involves glucose uptake and its conversion to pyruvate, then acetyl coenzyme A, fuelling the tricarboxylic acid (TCA) cycle. The TCA cycle produces a range of molecules, including nicotinamide adenine dinucleotide (NADH) which is used in the electron transport chain (ETC) and oxidative phosphorylation (OXPHOS), driving adenosine triphosphate (ATP) synthesis. This process is highly efficient but requires oxygen for the ETC to function properly. In hypoxic conditions, cells instead convert glucose-derived pyruvate to lactate using lactate dehydrogenase (LDH), sacrificing the efficiency of TCA/OXPHOS metabolism for rapid LDH-driven ATP production.

Rapidly proliferating cells typically exhibit aerobic glycolysis metabolism, preferentially converting glucose-derived pyruvate to lactate irrespective of oxygen availability, conferring proliferative advantages. Many factors contribute to the establishment of this altered metabolic state in tumour cells, including the expression of the M2 isoform of pyruvate kinase (PKM2) and aberrant activation of HIF1 signalling. Although such studies are limited, there is growing appreciation of the role of ncRNA EV cargo in reprogramming cellular metabolism.

A circRNA array in BCSC-derived EVs, identified *circCARM1* elevation in EVs from breast tumour spheroids compared to spheroids formed from adjacent normal tissue. These EVs promoted glycolysis in recipient MDA-MB-231 cells, whilst short hairpin RNA (shRNA)-mediated suppression of *circCARM1* abrogated the pro-glycolytic phenotype. *CircCARM1* interacts with *miR-1252-5p* in vitro and negatively correlates with *miR-1252-5p* in BC tissues, and expression of *circCARM1* promotes the upregulation of glycolytic enzyme 6-phosphofructo-2-kinase/fructose-2,6-biphosphatase 2 (PFKFB2) through *miR-1252-5p* sequestration [144].

Co-culturing TAMs and MDA-MB-231 BC cells in a transwell system increased glucose consumption and lactate production in MDA-MB-231 cells and elevated expression of glycolytic enzymes like PKM2 and LDHA, stabilising HIF1α. In this study, strong upregulation of 5 lncRNAs (*HISLA, LINC01234, LINC02432, LINC01678* and *LINC01493*) in an EV-secretion-dependent manner was validated by qRT-PCR and suppression of *HISLA* in TAMs abrogated the co-culture driven induction of aerobic glycolysis in BC cells. *HISLA* contributes to HIF1α activation through disrupting its interaction with prolyl hydroxylase domain-containing protein 2 (PHD2), leading to HIF1α stabilisation [138].

Similarly, *miR-503-3p* is upregulated in BC cell lines and tissues, correlating with more advanced disease [145]. *miR-503-3p* directly targets and suppresses dishevelled binding antagonist of beta catenin 2 (DACT2), activating WNT/β-catenin signalling and a glycolytic shift in metabolic activity. Importantly, M2 macrophage-derived EVs carry *miR-503-3p*, and recipient BC cells also exhibit metabolic changes, further highlighting how macrophage-derived EVs within the TME regulate tumour cell metabolism to promote growth [145].

One study found that uptake of CAF-derived EVs by BC cells impairs mitochondrial function and elevates extracellular acidification rate, suggesting increased aerobic glycolysis. Secretion of the lncRNA *SNHG3* was significantly higher from CAFs than normal breast MCF-10A cells, and treatment of MDA-MB-453 cells with EVs from *SNHG3*-suppressed CAFs reduced BC proliferation and restored mitochondrial metabolism. *SNHG3* suppresses *miR-330-5p* in BC cells leading to pyruvate kinase upregulation and increased glycolysis, which was validated in vivo using MDA-MB-453 tumours in mice [146].

Conversely, uptake of BC-derived EVs by CAFs leads to a MYC-dependent glycolytic increase, mediated by *miR-105*. Yan et al. showed that *miR-105* is carried by MDA-MB-231 EVs and directly targets MYC-antagonist MXI1. *miR-105* reprogrammes CAF metabolism in response to nutrient availability, promoting glycolysis and glutaminolysis in nutrient rich contexts to produce fuels for nearby cancer cells, or promoting detoxification of waste by-products lactic acid and ammonium in nutrient-starved conditions to enhance tumour cell survival. Orthotopic implantation of patient-derived BC xenografts with anti-*miR-105* expressing CAFs in a female Non-Obese Diabetic (NOD)/Severe Combined Immunodeficiency (SCID)/IL2Rγ-null (NSG) mouse, demonstrated that mice with *miR-105*-resistant CAFs exhibit significantly reduced tumour growth [147].

*miR-122* was shown to be highly secreted in EVs from MDA-MB-231 BC cells compared to MCF-10A, and BC-EV-carried *miR-122* suppressed glucose metabolism through downregulation of PKM2 expression in recipient cells. *miR-122* suppressed glucose uptake in lung fibroblasts and astrocytes, and EVs containing high levels of *miR-122* were shown to be taken up by lung and brain tissues in vivo. Notably, though overexpression of *miR-122* in xenograft tumours reduced primary tumour growth, these animals exhibited significantly increased metastasis to brain and lung tissues, where glucose availability was higher due to reduced local tissue utilisation [148].

Treatment of SCID mice with MDA-MB-231 BC EVs or EVs from *miR-122* overexpressing MCF-10A cells suppressed insulin signalling, inducing endogenous glucose production. Suppressed insulin signalling was attributed to reduced insulin secretion, due to PKM2 suppression in pancreatic islet β-cells following uptake of *miR-122-*carrying EVs. Knockout of *miR-122* in BC cells improved insulin signalling, lowered blood glucose levels and reduced tumour growth and proliferation, and corresponding results were seen in a patient-derived xenograft model. This demonstrates the regulation of β-cell insulin signalling by BC EVs, tying the effect to *miR-122*-mediated suppression of PKM2 by demonstrating that exogenous expression of PKM2 rescues the phenotype in the mouse model [149].

## EV ncRNA as a diagnostic biomarker in breast cancer

Current BC screening and diagnosis relies on mammography, ultrasound, MRI, and biopsy. Although these techniques have led to a reduction in BC mortality, they are invasive, costly, time-consuming, associated with false-negative results, and can lead to patient anxiety [150]. Additionally, the current investigations have limited spatial resolution which limits their ability to be used to determine the minimal residual disease status of patients following treatment. Therefore, the implementation of a novel diagnostic biomarker would be invaluable in BC diagnosis and monitoring.

**Fig. 3:**
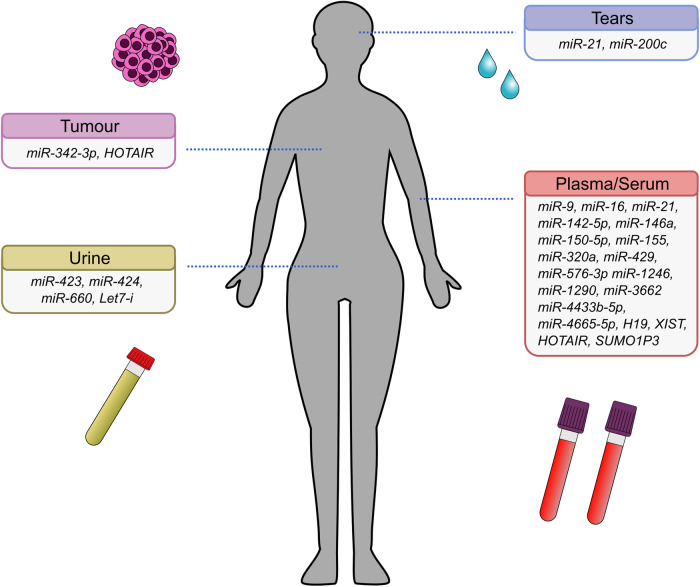
The diagnostic potential of ncRNAs in EVs in BC. The different ncRNAs which may be useful as biomarkers for breast cancer and the biofluids from which they were obtained.

The most well-studied EV ncRNA is miRNA. Key miRNAs with differing expression profiles include *miR-9*, *miR-16*, *miR-21*, *miR-429*, *miR-320a*, *miR-1246* and *miR-4433b-5p* which have been collected from serum, plasma, or urine EVs [151–153]. Furthermore, to improve their role as a biomarker, panels of miRNAs have been developed and shown to lead to a more accurate diagnosis, with a combination of *miR-21*, *miR-16*, *miR-9*, and *miR-429* enabling discrimination between different BC subtypes and healthy controls (sensitivity 96.8% and specificity 80.0%) [151] and a predictive model of 16 EV miRNAs outperforming routine diagnostic methods [154] (Fig. 3).

Other studies have also investigated EVs from other biofluids including urine and tears. Schirmer tear test strips have been used to show *miR-21* and *miR-200c* were significantly higher in BC patient EVs compared to healthy controls [155]. Another study tested a miRNA panel of *miR-424*, *miR-423*, *miR-660*, and *let7-i* on EVs isolated from urine of recently diagnosed BC patients and healthy controls which showed 98.6% sensitivity and 100% specificity [156].

Screening miRNAs in EV serum samples of BC patients and healthy controls identified *miR-142-5p*, *miR-320a*, and *miR-4433b-5p* as differentially expressed [157]. Collectively, these miRNAs differentiate between various tumour characteristics, including subtype, size and grade. Significantly higher expression of *miR-150-5p* (AUC = 0.705), *miR-576-3p* (AUC = 0.691), and *miR-4665-5p* (AUC = 0.681) in plasma EVs from TNBC patients was shown to be a potential biomarker for recurrence [158]. Furthermore, EVs can be separated from tumour tissues removed during surgery to inspect *miR-342-3p* levels which correlate with chemo-resistance [143]. Another study found *miR-124*6 (AUC = 0.750, 78.1% sensitivity and 75% specificity) and *miR-155* (AUC = 0.877, 68.8% sensitivity and 97.2% specificity) were significantly upregulated in EVs isolated from blood samples from trastuzumab-resistant compared to trastuzumab-sensitive BC patients [159].

Some EV lncRNAs also demonstrate potential as diagnostic markers for BC such as *H19* [137, 160], *XIST* [161], and *HOX transcript antisense* *RNA* (*HOTAIR*) [162]. BC cells upregulate *HOTAIR* compared to adjacent tissues and healthy controls, whilst serum samples of BC patients showed increased levels of *HOTAIR* in EVs compared to healthy controls. Increased *HOTAIR* levels correlate with shorter survival, poor response to therapies [163] and HER2 positivity [164].

BC patients, especially those with TNBC, have higher levels of lncRNA *SUMO1P3* in tissue and serum-derived EVs compared to healthy controls [165]. In this study, increased serum EV *SUMO1P3* positively correlated with increased invasion, metastasis, and worse overall survival. After chemotherapy, *SUMO1P3* levels decreased significantly in chemo-sensitive patients, but remained unchanged in chemo-resistant patients, highlighting *SUMO1P3* as a potential prognostic EV biomarker for BC. Interestingly, *SUMO1P3* negatively regulates *miR-320a* [166] which has tumour suppressive qualities and is found in the EVs of patients with smaller, early-stage BC tumours [157]. Therefore, a higher level of *SUMO1P3* combined with a lower level of *miR-320a* could be used in combination as a prognostic marker for more aggressive BC tumours (Table 2).

**Table 2:** Summary of the diagnostic potential of ncRNAs in EVs.

ncRNA(s)	EV source	Diagnosis	Diagnostic Value	Reference
*miR-9*	Plasma	BC screening	AUC = 0.71	[151]
*miR-16*	Plasma	BC screening	AUC = 0.85	[151]
*miR-21*	Plasma	BC screening	AUC = 0.70	[151]
	Urine	BC screening	70.8% Sensitivity and 78.3% specificity	[167]
	Plasma	BC screening	AUC = 0.69	[153]
	Tears	BC screening	–	[155]
*miR-429*	Plasma	BC screening	AUC = 0.71	[151]
*miR-21, miR-16, miR-9, and miR-429*	Plasma	BC screening	AUC = 0.88, 96.8% sensitivity and 80% specificity	[151]
		Luminal A	AUC = 0.90	
		Luminal B	AUC = 0.86	
		HER-2	AUC = 0.88	
		Triple-negative	AUC = 0.84	
*miR-320a*	Serum	BC screening	AUC = 0.806, 93.3% sensitivity and 68.75% specificity	[157]
*miR-320a, miR-142-5p, miR-4433b-5p*	Serum	BC screening	AUC = 0.839, 93.55% sensitivity and 68.755 Specificity	[157]
*miR-1246*	Blood	trastuzumab-resistant HER-2 patients	AUC = 0.750, 78.1% sensitivity and 75% specificity	[159]
	Plasma	BC screening	AUC = 0.69	[153]
*miR-21 and miR-1246*	Plasma	BC screening	AUC = 0.73	[153]
*miR-200c*	Tears	BC screening	–	[155]
*miR-424*	Urine	BC screening	AUC = 0.88	[156]
*miR-423*	Urine	BC screening	AUC = 0.86	[156]
*miR-660*	Urine	BC screening	AUC = 0.85	[156]
*Let7-i*	Urine	BC screening	AUC = 0.87	[156]
*miR-424, miR-423, miR-660, and let7-i*	Urine	BC screening	98.6% sensitivity and 100% specificity	[156]
*miR-3662, miR-146a, miR-1290*	Serum	Lymph node metastasis, surgery and chemotherapy monitoring	–	[168]
*miR-342-3p*	Cancerous Tissue	Chemo-resistance	–	[143]
*miR-150-5p*	Plasma	Recurrence	AUC = 0.705	[158]
*miR-576-3p*	Plasma	Recurrence	AUC = 0.691	[158]
*miR-4665-5p*	Plasma	Recurrence	AUC = 0.681	[158]
*miR-155*	Blood	trastuzumab-resistant HER-2 patients	AUC = 0.877, 68.8% sensitivity and 97.2% specificity	[159]
lncRNA *H19*	Serum	BC screening	AUC = 0.870, 87.0% sensitivity and 70.6% specificity	[160]
	Serum	DOX-resistant patients	AUC = 0.752	[137]
			75% sensitivity and 65.2% specificity	
lncRNA *XIST*	Serum	TNBC patients	AUC = 0.888	[161]
lncRNA *HOTAIR*	Serum	BC screening	AUC = 0.918	[163]
	Cancerous Tissue	Disease-free survival	worse disease-free survival (P = 0.0481)	[163]
		Overall survival	And overall survival (P = 0.0463)	
lncRNA *SUMO1P3*	Serum	TNBC patients	–	[165]

To provide clinical utility, EV based biomarkers will need to demonstrate improvements compared to the internationally accepted standard of care investigations. The current studies investigating the use of EV ncRNA as biomarkers in BC have been focussed on discovery and initial technical validation. To progress further, researchers will need to perform much larger studies focussing on identifying the optimal role for these novel biomarkers in BC diagnostics and monitoring. These studies would need to be designed carefully and with appropriate power to demonstrate superior diagnostic ability compared to current investigations, either through improved patient acceptability or increased sensitivity. Alternatively, they could be studied as an additional test to the standard of care investigations to further reduce the false positive and false negative rates. Given the non-invasive nature of such proposed tests, it is likely these methods would demonstrate significant improvements in patient acceptability, though the relative sensitivity remains to be seen. With around 30 biofluids in humans, including saliva, ascites and lymph all potentially acting as reservoirs for cancer-derived EVs, the field of EV ncRNA biomarker discovery for BC is still in its early stages and the possibility of finding and implementing a highly specific and sensitive diagnostic, prognostic or predictive panel of ncRNA biomarkers for BC still remains [5].

Despite the identification of numerous promising biomarker ncRNAs in EVs for BC, significant challenges remain, particularly those associated with the separation of EVs from biofluids and their subsequent analysis. EV isolation is a technically challenging procedure that can be costly, time-consuming and highly variable with different protocols enriching different EV subpopulations [5]. As such, the detection of rare EV subpopulations in biofluids will need to be suitably robust to meet the requirements for large-scale, reliable diagnostics.

## Conclusions

A vast number of studies have demonstrated the importance of ncRNAs in EV-mediated communication between cancer cells and the TME. Various forms of ncRNA have been linked to diverse processes in BC progression, including invasion and metastasis, growth, angiogenesis, metabolic changes, and drug resistance. Targeting pathways relevant to the regulatory role of ncRNAs in the TME may therefore represent a useful strategy for BC treatment. As a biomarker, EV-contained ncRNA could be useful for diagnosis, monitoring and predicting therapy responses due to the high specificity and sensitivity of ncRNAs, particularly combined as panels. Less invasive diagnostic techniques are preferable due to increased cooperation from patients, and so the further development and implementation of ncRNA-based diagnostic techniques could therefore increase early detection of BC, leading to improved survival.
